# Diabetes as a risk factor of death in hospitalized COVID-19 patients – an analysis of a National Hospitalization Database from Poland, 2020

**DOI:** 10.3389/fendo.2023.1161637

**Published:** 2023-05-04

**Authors:** Michal Kania, Beata Koń, Konrad Kamiński, Jerzy Hohendorff, Przemysław Witek, Tomasz Klupa, Maciej T. Malecki

**Affiliations:** ^1^ Department of Metabolic Diseases and Diabetology, Jagiellonian University Medical College, Krakow, Poland; ^2^ Department of Metabolic Diseases and Diabetology, University Hospital, Krakow, Poland; ^3^ Department of Analysis and Innovation, National Health Fund, Warsaw, Poland

**Keywords:** COVID-19, diabetes, mortality, epidemiology, modelling, propensity-score matching

## Abstract

**Introduction:**

Diabetes is one of the comorbidities associated with poor prognosis in hospitalized COVID-19 patients. In this nationwide retrospective study, we evaluated the risk of in-hospital death attributed to diabetes.

**Methods:**

We analyzed data from discharge reports of patients hospitalized with COVID-19 in 2020 as submitted to the Polish National Health Fund. Several multivariate logistic regression models were used. In each model, in-hospital death was estimated with explanatory variables. Models were built either on the whole cohorts or cohorts matched with propensity score matching (PSM). The models examined either the main effects of diabetes itself or the interaction of diabetes with other variables.

**Results:**

We included 174,621 patients with COVID-19 who were hospitalized in the year 2020. Among them, there were 40,168 diabetic patients (DPs), and the proportion of DPs in this group was higher than in the general population (23.0% vs. 9.5%, p<0.001). In this group of COVID-19 hospitalizations, 17,438 in-hospital deaths were recorded, and the mortality was higher among DPs than non-diabetics (16.3% vs. 8.1%, p<0.001). Multivariate logistic regressions showed that diabetes was a risk factor of death, regardless of sex and age. In the main effect analysis, odds of in-hospital death were higher by 28.3% for DPs than for non-diabetic patients. Similarly, PSM analysis including 101,578 patients, of whom 19,050 had diabetes, showed that the risk of death was higher in DPs regardless of sex with odds higher by 34.9%. The impact of diabetes differed among age groups and was the highest for patients aged 60-69.

**Conclusions:**

This nationwide study confirmed that diabetes was an independent risk factor of in-hospital death in the course of COVID-19 infection. However, the relative risk differed across the age groups.

## 1 Introduction

Coronavirus disease-2019 (COVID-19) caused by the severe acute respiratory syndrome coronavirus-2 (SARS-CoV-2), emerged at the end of 2019 and caused a global pandemic (1). The first case of COVID-19 in Poland was identified on March 3, 2020. This was followed by the first wave of the pandemic and triggered the first national lockdown that lasted approximately until the end of June 2020. The second wave started at the beginning of August and ended in January 2021. According to official data from the Polish government, a total of 68,505 excess deaths were recorded in 2020, which accounts for a 16% increase in total mortality compared to the period 2017-2019 (2). The challenges concerning preparation of the healthcare system to properly respond to this new epidemiological threat, required urgent action. The COVID-19 pandemic profoundly impacted the healthcare system in Poland. The Polish Ministry of Health initially decided to create a network of hospitals dedicated exclusively to serve as a multispecialty reference center for COVID-19 patients (3, 4). This model was modified in October 2020 when temporary COVID-19 hospitals were established and numerous regional hospitals were converted in order to play a supportive role in managing COVID-19 patients (5). Until the end of July 2022, there were 6,050,000 cases of SARS-Cov-2 infections in Poland with deaths totaling around 115,500 (6).

Numerous factors, such as male gender, age, diabetes, obesity, or cardiovascular disease, have been identified as risk factors for hospitalization of COVID-19 patients (7–10). Diabetes, along with hypertension, cardiovascular and respiratory diseases, is one of the most common comorbidities of COVID-19. It has been associated with a higher risk of unfavorable COVID-19 outcomes, such as longer hospitalization, higher in-hospital mortality, ICU admission and requirement for mechanical ventilation (11–14). However, most original studies reporting this association were retrospective single-center reports. There are few analyses of datasets from large healthcare initiatives or from the nationwide level of COVID-19 patients (15, 16).

In this retrospective study, we evaluated diabetes as a risk factor for in-hospital mortality using data from the Polish National Health Fund, a state institution that finances healthcare services from contributions paid by the insured persons (17) and runs a nationwide healthcare database.

## 2 Methods

For this retrospective analysis, information concerning patients hospitalized between the 1^st^ Jan and 31^st^ Dec 2020 due to COVID-19 was extracted from data reported to the Polish National Health Fund. Analyzed data included hospitalizations that were financed from the governmental COVID-19 Counteracting Fund and included information on both insured and uninsured patients. If the patient was hospitalized more than once in 2020, then the first hospitalization of the patient was included in the study. Only adult patients were included in the study defined as people aged at least 17 years old as of Dec 31^st^ 2019.

Hospital information from discharge charts of COVID-19 patients was merged with data from diabetes databases and data regarding comorbidities from reports delivered to the National Health Fund that health care providers sent to receive reimbursement for health care benefits delivered due to other health problems than COVID-19. They included medical benefits reported with a specific ICD-10 diagnosis code (either as a main or additional diagnosis), or ICD-9 procedure code in the case of hemodialysis, if the patient had it within the last 3 years. The list included diabetes, obesity, loss of weight, cardiac arrhythmias, arterial hypertension, heart failure, peripheral artery disease, dyslipidemia, ischemic heart disease, history of myocardial infarction and stroke, cardiomyopathy, valvular disorders, atrial fibrillation, pulmonary circulation disorders, chronic pulmonary diseases, chronic kidney disease, hypothyroidism and hyperthyroidism, other thyroid diseases, liver disorders, coagulopathies, anemia, electrolyte imbalances, history of neoplasm, rheumatoid diseases, paralysis, other neurological disorders, depression, drug abuse, and psychosis. The full list with corresponding ICD codes is presented in **Table S1** in the Appendix.

Separately, for diabetic patients (DPs) and patients without diabetes, characteristics such as mean age, proportion of population older than 65, proportion of population older than 85, sex and in-hospital death were compared. For each of these variables, we examined whether differences between diabetic and non-diabetic populations were statistically significant. For age, Welch two sample t-test was used, and for other characteristics, referring to share of population, a chi-square test was used.

### 2.1 Logistic regression modelling

Econometric modelling was used to analyze the impact of multiple variables on the risk of in-hospital death of COVID-19 patients. Four different multivariate logistic regression models were estimated. In each model, a dependent variable was binary in nature describing if a patient’s hospitalization ended at the same day, when the patient died. Explanatory variables included diagnosis of diabetes, as well as additional confounders: sex, logarithm of age (recorded as difference between 2020 and year of patient’s birth) and the diagnosis of the selected comorbidities (named above). The first two models were built for the whole cohort, another two for the matched cohort using propensity score matching (PSM). All models were estimated with the same set of explanatory variables, however in the first and third model only main effects were included. In the second and fourth model, interactions between diabetes and other variables were also included.

PSM was applied, as diabetes can affect probability of being hospitalized due to COVID-19 causing a non-random sample. Each DP was matched with non-diabetic patient(s) according to other 36 variables (sex, age group 18-24, 25-29, 30-34,…, 85-89, 90-94, 95+, comorbidities other than diabetes as shown in **Table S1**, i.e. arterial hypertension, other neoplasms, health failure, cardiac arrhythmias etc.). One-to-many exact matching was used (18), i.e. non-diabetic patient or patients were matched to each singular DP (according to above mentioned parameters). The number of matched non-diabetics was not limited. If DP was not matched with non-diabetic patient, their data was discarded from the analysis.

In order to minimize the number of variables and include only those with strong effect on the in-hospital death, the following approach to select variables in the final models for each of four setting was used: the dataset was divided into train (80% of observations) and test dataset (20% of observations). In the first step, a model only with the logarithm of age was built. Then, for each explanatory variable, a model with the logarithm of age and that variable was built. Furthermore, each of those models was compared with the model containing only the logarithm of age and Bayes Factor (BF) was calculated (19). If the maximum value of BF was greater than 30, then the variable with the highest BF was added to the final model. Subsequently, the procedure was repeated, and a model with the logarithm of age and selected variable was built; each of the remaining variables was separately added to the model, and for each model BF was calculated. If the maximum BF was greater than 30, then the variable was added to the model and the procedure was repeated until maximum BF for remaining variables was above 30.

The models’ estimates were used to calculate the probability of in-hospital death according to the patient’s age, sex and diagnosis of diabetes. The quality of the models was tested on a test dataset and final models were estimated on the whole dataset. To assess goodness of fit, McFadden’s pseudo R^2^ was used. Furthermore, the quality of the models was verified with area under ROC curve (AUC) calculated on the test set.

All the analyses were conducted with SPSS Pro 6.0 and in R, version 3.6.1 (20, 21). For statistical significance a threshold of 0.05 for p-value was used.

## 3 Results

The study included a total of 174 621 COVID-19-infected patients who were hospitalized in Poland between the 1^st^ Jan and 31^st^ Dec 2020. The mean age was 60.5 ± 18.9 years and the median age was 63 years (IQR 29). The median age in diabetic patients was 72 (IQR 16) and 59 (IQR 31) in non-diabetic patients. There were 90 628 (51.9%) men in the study group (**Table 1**). The estimated number of patients with diabetes in Poland in 2018 was 2,864,000 (approximately 9.1% of adult population according to official registries (22)). Of 174 621 patients hospitalized with COVID-19 infection, 40 168 had diabetes (23.0%) and it was more common than in the general population (p<0.001). The prevalence of comorbidities, including but not limited to cardiovascular, metabolic, pulmonary, psychiatric and endocrine disorders, was higher in DP than in non-diabetic COVID-19 patients (**Table 2**). 17 438 in-hospital deaths were recorded (10.0%), the mortality was higher in DPs, compared to non-diabetics (16.3% vs. 8.1%, p<0.001). It was higher across all age groups regardless of patients gender and age (**Table S2**).

**Table 1:** Basic characteristics of DPs and non-diabetic patients with in-hospital death data.

Characteristics	All	Diabetes	Non-diabetes	P value
Number	174 621	40 168 (23%.0)	134 453 (77.0%)	–
Age [years (SD)]	60.5 (18.92)	70.9 (12.58)	57.4 (19.39)	<0.001
Age > 65 years [n (%)]	113 643 (65.1%)	29 927 (74.5%)	53 789 (40%)	<0.001
Age > 85 years [n (%)]	21 679 (12.4%)	5 094 (12.7%)	11 491 (8.5%)	<0.001
Male [n (%)]	90 628 (51.9%)	21 113 (52.6%)	69 515 (51.7%)	0.002
Endpoint				
In-hospital death [Yes (%)]	17 438 (10.0%)	6 541 (16.3%)	10 897 (8.1%)	<0.001

**Table 2:** The prevalence of comorbidities in the hospitalized population.

Comorbidities	Non-diabetic	Diabetic	P value
Number	134 453 (77.0%)	40 168 (23%.0)	-
Arterial hypertension	60 742 (45.2%)	34 363 (85.6%)	<0.001
Neoplasm. Other	26 445 (19.7%)	9 366 (23.3%)	<0.001
Heart failure	19 843 (14.8%)	15 286 (38.1%)	<0.001
Cardiac arrhythmias	22 871 (17.0%)	12 216 (30.4%)	<0.001
Dyslipidaemia	21 237 (15.8%)	13 283 (33.1%)	<0.001
Ischemic heart disease	18 708 (13.9%)	14 446 (36.00%)	<0.001
Chronic pulmonary disorders	19 826 (14.8%)	8 406 (20.9%)	<0.001
Peripheral artery disease	15 029 (11.2%)	9 887 (24.6%)	<0.001
Atrial fibrillation	13 189 (9.8%)	8 615 (21.5%)	<0.001
Neoplasm. malignant	14 212 (10.6%)	6 278 (15.6%)	<0.001
Anaemia	12 096 (9.0%)	6 207 (15.5%)	<0.001
Hypothyroidism	11 010 (8.2%)	4 712 (11.7%)	<0.001
Chronic kidney disease	7 409 (5.5%)	7 253 (18.1%)	<0.001
Depression	10 104 (7.5%)	3 227 (8.0%)	<0.001
Other neurological disorders	8 481 (6.3%)	2 911 (7.3%)	<0.001
History of stroke	6 500 (4.8%)	4 013 (10.0%)	<0.001
Angina	5 227 (3.9%)	4 002 (10.0%)	<0.001
Electrolyte imbalances	5 552 (4.1%)	3 023 (7.5%)	<0.001
Alcohol abuse	7 104 (5.3%)	1 377 (3.4%)	<0.001
Obesity	3 600 (2.7%)	4 039 (10.1%)	<0.001
Liver disorders	4 361 (3.2%)	2 145 (5.3%)	<0.001
Valvular disorders	4 172 (3.1%)	2 331 (5.8%)	<0.001
Rheumatoid diseases	4 527 (3.4%)	1 627 (4.1%)	<0.001
History of myocardial infarction	2 974 (2.2%)	2 536 (6.3%)	<0.001
Other thyroid disorders	3 874 (2.9%)	1 237 (3.1%)	0.03
Paralysis	2 857 (2.1%)	1 473 (3.7%)	<0.001
Psychosis	3 198 (2.4%)	847 (2.1%)	0.002
Hyperthyroidism	2 350 (1.8%)	1 038 (2.6%)	<0.001
Dialysis	1 680 (1.3%)	1 410 (3.5%)	<0.001
Pulmonary circulation disorders	1 996 (1.5%)	846 (2.1%)	<0.001
Coagulopathies	1 767 (1.3%)	735 (1.8%)	<0.001
Cardiomyopathy	1 425 (1.2%)	966 (2.4%)	<0.001
Drug abuse	1 897 (1.4%)	355 (0.9%)	<0.001
Weight loss	1 746 (1.3%)	491 (1.2%)	0.23

**Figure 1:**
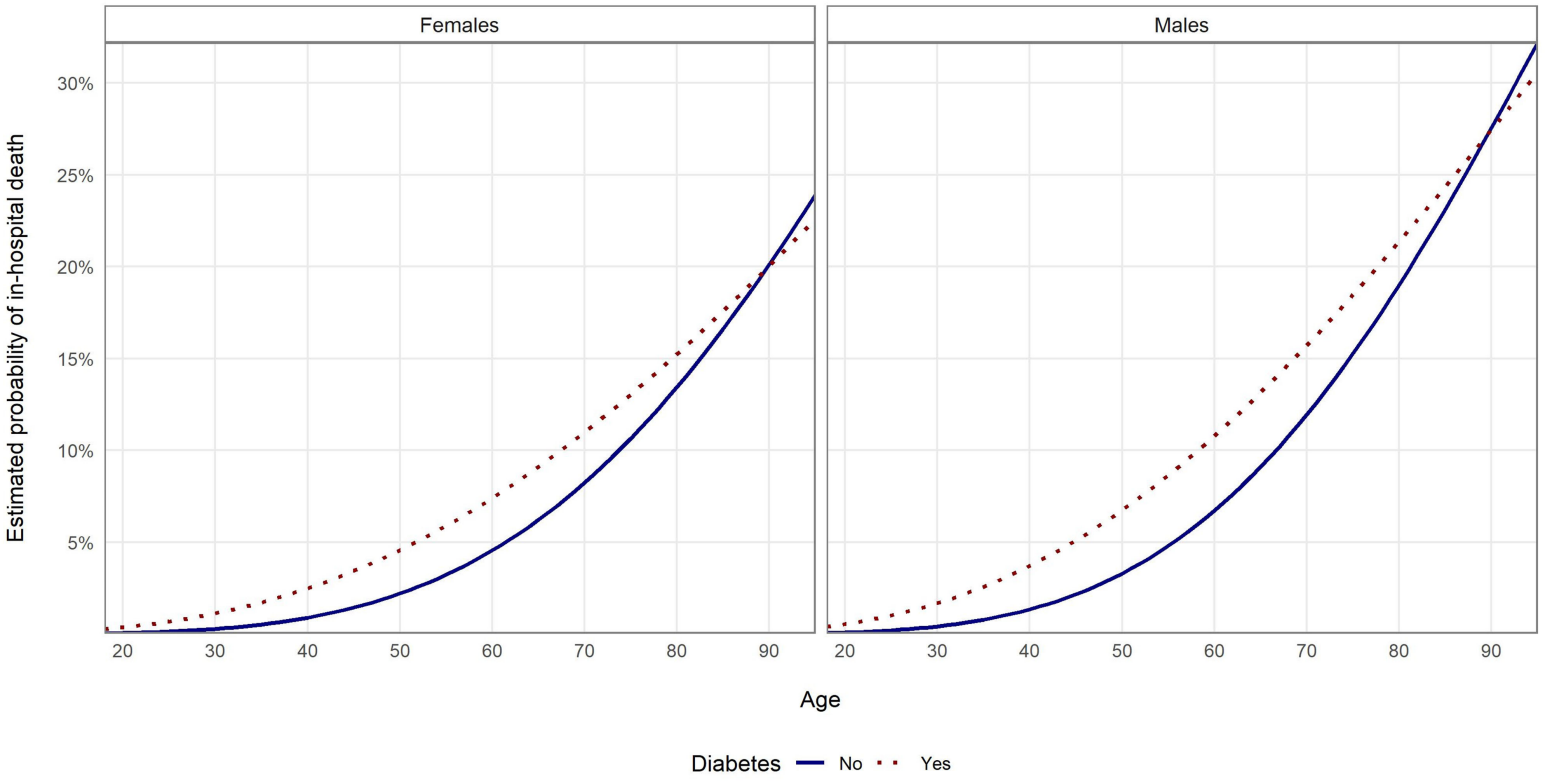
Probability of in-hospital death in the examined groups according to age and sex estimated with multivariate logistic regression without PSM and with interaction analysis for patients without other comorbidities.

**Figure 2:**
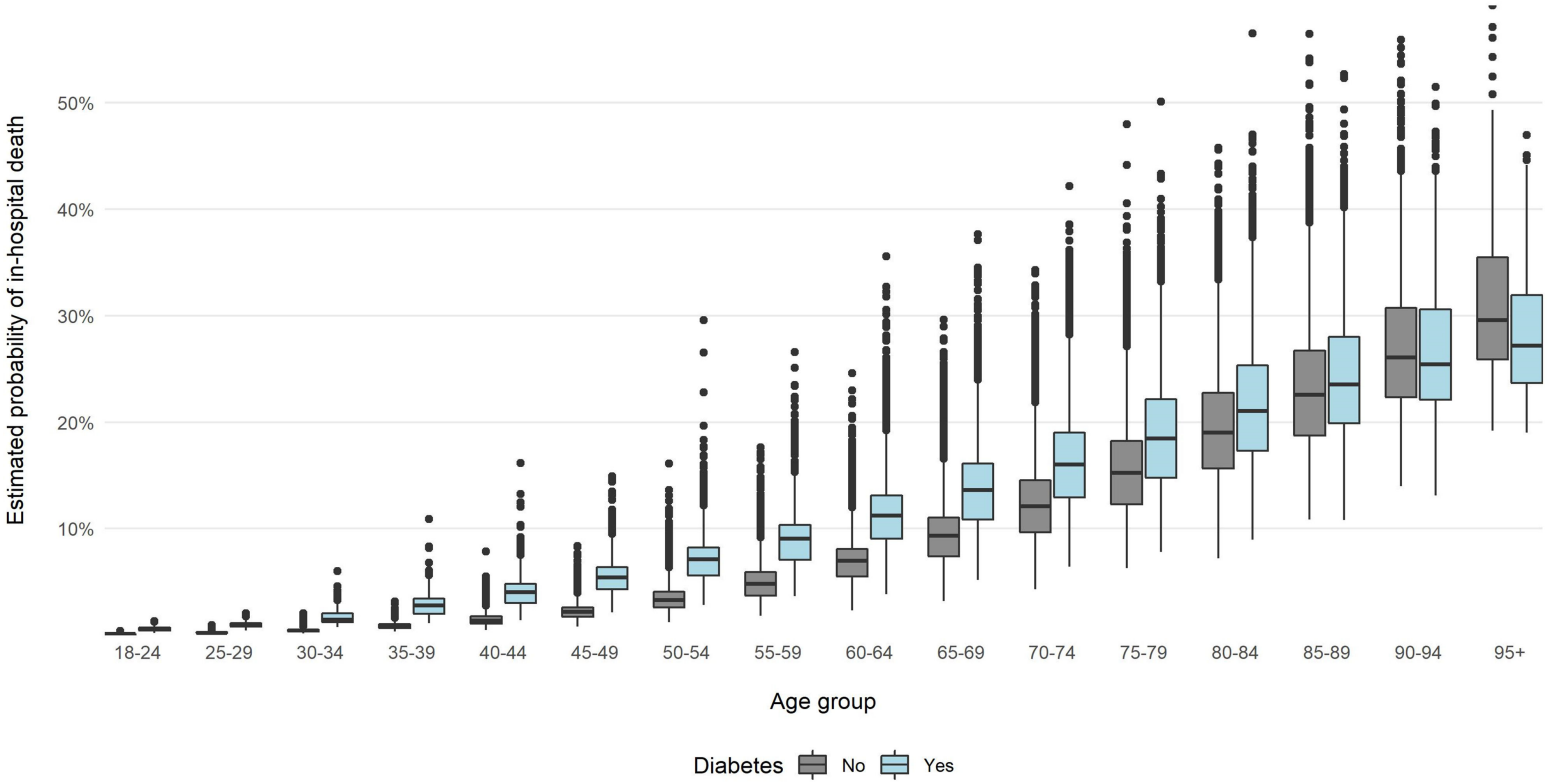
Probability of in-hospital death in the examined groups according to age and sex estimated with multivariate logistic regression without PSM and with interaction analysis.

Results of multivariate logistic regression (**Table 3**) showed that diabetes and some other comorbidities, including chronic kidney disease, heart failure, chronic ischemic heart disease, cardiac arrythmias and neoplasms, were associated with a higher risk of death, regardless of sex and age. The risk was higher in older patients and in males. For patients with diabetes, odds of in-hospital death were higher by 28.3% than for patients without diabetes. The regression model that included interactions of variable referring to diabetes with other variables, showed that impact of diabetes on COVID-19 in-hospital death was influenced by the patient’s age (**Table S3**). Results of the model are visualized on **Figures 1**; **2**. **Figure 1** shows probability of in-hospital death according to patients’ sex, age and the fact of being diabetic without other comorbidities. **Figure 2** shows probability of in-hospital death according to patients’ sex, age and fact of being diabetic for analyzed group of patients. Results show that the highest difference in median value of COVID-19 in-hospital death between diabetic and non-diabetic patients was for patients in age group 65-69 (4.3 percent point), 60-64 and 55-59 (4.2 percent point) (**Table S3**).

**Table 3:** Results of logistic regression modelling for the risk of in-hospital death.

Variable	Model without interactions of variable diabetes with other variables			Model with interactions of variable diabetes with other variables		
	Estimate (SE)	Odds ratio (95% CI)	P-value	Estimate (SE)	Odds ratio (95% CI)	P-value
Intercept	-18.714 (0.208)	0.000 (0.000 – 0.000)	<0.001	-19.845 (0.236)	0.000 (0.000 – 0.000)	<0.001
log(Age)	3.838 (0.049)	46.414 (42.179 - 51.075)	<0.001	4.104 (0.055)	60.560 (54.334 - 67.5)	<0.001
Gender: male	0.425 (0.017)	1.529 (1.478 - 1.581)	<0.001	0.413 (0.017)	1.511 (1.460 - 1.563)	<0.001
Diabetes	0.249 (0.018)	1.283 (1.238 - 1.328)	<0.001	5.787 (0.46)	325.885 (132.16 - 803.578)	<0.001
Chronic kidney disease	0.218 (0.026)	1.243 (1.181 - 1.308)	<0.001	0.228 (0.026)	1.256 (1.193 - 1.322)	<0.001
Other neurological disorders	0.299 (0.029)	1.348 (1.273 - 1.428)	<0.001	0.299 (0.029)	1.348 (1.273 - 1.428)	<0.001
Heart failure	0.245 (0.020)	1.278 (1.228 - 1.329)	<0.001	0.247 (0.02)	1.280 (1.231 - 1.332)	<0.001
Neoplasm, malignant	0.257 (0.024)	1.293 (1.235 - 1.354)	<0.001	0.257 (0.023)	1.293 (1.235 - 1.354)	<0.001
Psychosis	0.391 (0.053)	1.478 (1.332 - 1.640)	<0.001	0.375 (0.053)	1.456 (1.312 - 1.615)	<0.001
Cardiac arrhythmias	-0.126 (0.02)	0.881 (0.847 - 0.917)	<0.001	-0.121 (0.02)	0.886 (0.851 - 0.921)	<0.001
Haemodialysis	0.368 (0.052)	1.445 (1.304 - 1.601)	<0.001	0.337 (0.052)	1.401 (1.264 - 1.552)	<0.001
Neoplasm, other	-0.156 (0.022)	0.856 (0.82 - 0.893)	<0.001	-0.159 (0.022)	0.853 (0.817 - 0.891)	<0.001
Weight loss	0.363 (0.058)	1.438 (1.284 - 1.610)	<0.001	0.357 (0.058)	1.430 (1.277 - 1.601)	<0.001
Coagulopathies	0.331 (0.061)	1.392 (1.234 - 1.570)	<0.001	0.312 (0.061)	1.366 (1.211 - 1.540)	<0.001
Other thyroid disorders	-0.313 (0.061)	0.731 (0.649 - 0.823)	<0.001	-0.321 (0.061)	0.726 (0.644 - 0.817)	<0.001
Chronic ischemic heart disease	-0.092 (0.020)	0.912 (0.877 - 0.948)	<0.001	-0.094 (0.020)	0.910 (0.876 - 0.946)	<0.001
Anaemia	0.105 (0.024)	1.111 (1.059 - 1.164)	<0.001	0.106 (0.024)	1.112 (1.060 - 1.165)	<0.001
D:log(Age)*	–	–	–	-1.258 (0.107)	0.276 (0.224 - 0.341)	<0.001
*D: - interaction with diabetes	Train dataset – McFadden’s pseudo-R2: 0.138 Test dataset – AUC (area under ROC curve): 0.770 Complete dataset – McFadden’s pseudo-R2: 0.138			Train dataset – McFadden’s pseudo-R2: 0.139 Test dataset – AUC (area under ROC curve): 0.771 Complete dataset – McFadden’s pseudo-R2: 0.139		

**Figure 3:**
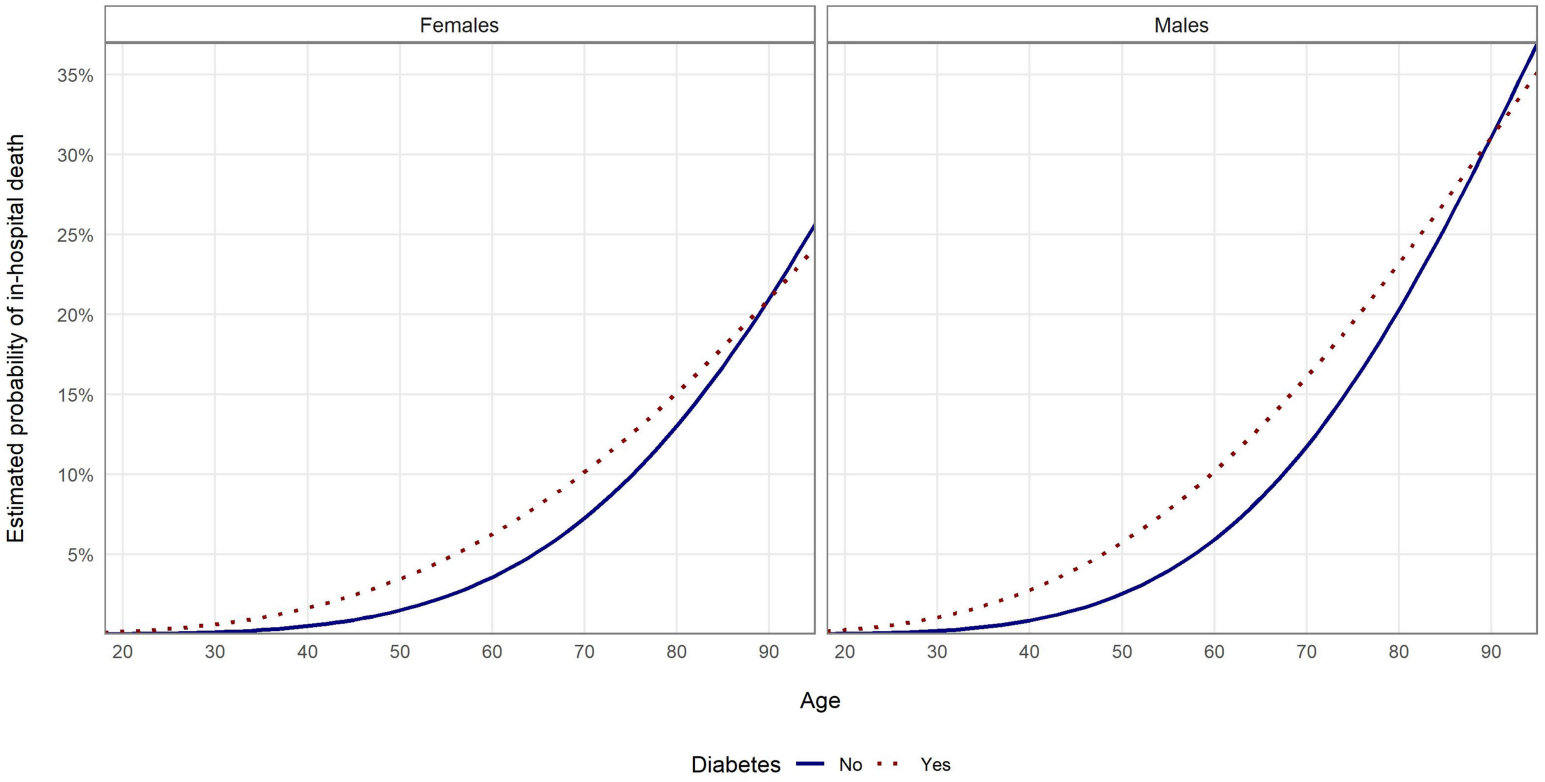
Probability of in-hospital death in the examined groups according to age and sex estimated with multivariate logistic regression with PSM and with interaction analysis for patients without other comorbidities.

**Figure 4:**
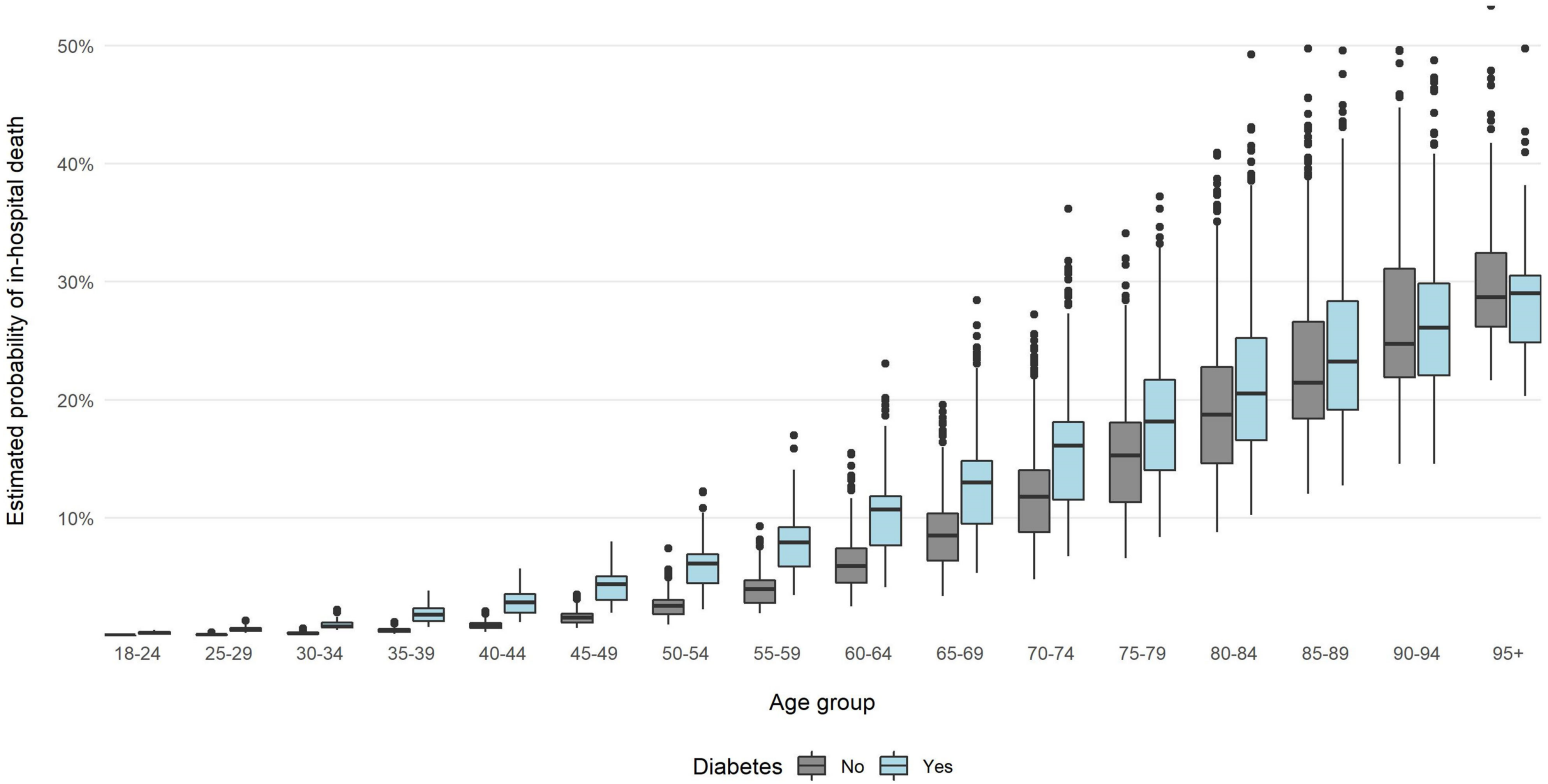
Probability of in-hospital death in the examined groups according to age and sex estimated with multivariate logistic regression with PSM and with interaction analysis.

For PSM, data from 101 578 patients was analyzed, of whom 19 050 had diabetes (i.e. 21 118 diabetic were not matched to non-diabetic patients). The characteristics of population matched by PSM is shown in **Tables S4**—**S6** in the Appendix. In the group of patients matched with PSM, the risk of COVID-19 in-hospital death was higher in DPs than for non-diabetic patients regardless of sex and age. Results of the multivariate logistic regression model showed that the odds for in-hospital death were 72.5% higher for men than for women (**Table 4**). For DPs, odds of death were higher by 34.9% than for non-diabetic patients. After adding interactions to the model, it showed similar results to the model estimated without PSM, i.e. age differentiated impact of the diabetes on the in-hospital risk of death (**Table 4**). Results of the models for patients without other comorbidities are presented on **Figure 3**. **Figure 4** presents values of estimated COVID-19 in-hospital death according to patients’ age group and fact of being diabetic for the analyzed group of patients. The median values are additionally presented in **Table S7**. The results show that the median value of the probability of in-hospital death was highest for patients aged 95 and more. The highest difference (in absolute values) between diabetic and non-diabetic patients was observed for age groups 60-64 (4.8 percent point) and 65-69 (4.5 percent point).

**Table 4:** Results of logistic regression modelling after PSM analysis for the risk of in-hospital death.

Variable	Model without interactions of variable diabetes with other variables			Model with interactions of variable diabetes with other variables		
	Estimate (SE)	Odds ratio (95% CI)	P-value	Estimate (SE)	Odds ratio (95% CI)	P-value
Intercept	-21.926 (0.317)	0.000 (0.000 – 0.000)	<0.001	-23.170 (0.358)	0.000 (0.000 – 0.000)	<0.001
log(Age)	4.561 (0.074)	95.716 (82.78 - 110.675)	<0.001	4.855 (0.084)	128.335 (108.918-151.214)	<0.001
Gender: male	0.545 (0.026)	1.725 (1.638 - 1.817)	<0.001	0.530 (0.026)	1.699 (1.614-1.789)	<0.001
Diabetes	0.300 (0.027)	1.349 (1.28 - 1.422)	<0.001	6.526 (0.694)	682.533 (175.129-2660.04)	<0.001
Chronic kidney disease	0.305 (0.056)	1.357 (1.215 - 1.515)	<0.001	0.319 (0.056)	1.375 (1.232-1.535)	<0.001
Other neurological disorders	0.420 (0.068)	1.523 (1.333 - 1.739)	<0.001	0.424 (0.068)	1.529 (1.338-1.746)	<0.001
Neoplasm, malignant	0.329 (0.045)	1.390 (1.273 - 1.518)	<0.001	0.328 (0.045)	1.389 (1.272-1.516)	<0.001
Neoplasm, other	-0.220 (0.042)	0.803 (0.739 - 0.871)	<0.001	-0.223 (0.042)	0.800 (0.737-0.868)	<0.001
Heart failure	0.233 (0.034)	1.263 (1.181 - 1.351)	<0.001	0.247 (0.034)	1.280 (1.197-1.369)	<0.001
Cardiac arrhythmias	-0.230 (0.038)	0.795 (0.738 - 0.856)	<0.001	-0.221 (0.038)	0.802 (0.745-0.864)	<0.001
D:log(Age)*	–	–	–	-1.450 (0.162)	0.234 (0.171-0.322)	<0.001
*D: - interaction with diabetes	Train dataset – McFadden’s pseudo-R2: 0.173 Test dataset – AUC (area under ROC curve): 0.807 Complete dataset – McFadden’s pseudo-R2: 0.172			Train dataset – McFadden’s pseudo-R2: 0.174 Test dataset – AUC (area under ROC curve): 0.808 Complete dataset – McFadden’s pseudo-R2: 0.174		

## 4 Discussion

In this report we present the analysis of a nationwide cohort of patients hospitalized due to COVID-19 in 2020. Of note, our study is one of the very few assessing the prevalence of COVID-19 hospitalizations and mortality on the nationwide level, with most studies to date focusing on the case series or hospital-based cohort of patients. We confirmed that diabetes was an independent risk of hospitalization and in-hospital death in the course of COVID-19, and provided also some new observations that are discussed below.

Results of our unadjusted analysis showed that the hospitalization rate was almost 2.5 times higher for DP than for non-diabetics. Most studies, to date, mainly up to mid-2021, reported a higher frequency of hospitalization due to COVID-19 (7–10, 23), with the risk being up to 4 times higher in DPs (23). After adjustment for age, gender, and co-existing comorbidities, the risk decreased, but in most studies was still statistically significant (7, 8, 10). In cohorts of the European origin, the risk for hospitalization in DPs was ca. twice as high as in population without diabetes (10, 24). Importantly, our data covers the period of the first two waves of COVID-19 pandemic in Poland. These numbers should be seen in the context that initially, due to a mandatory supervision by epidemiological services or hospitalization related to COVID-19, the number of patients admitted to the hospitals was disproportionately higher, as hospitalization of all patients with SARS-CoV-2 infection was obligatory (25). Of note, the proportion of DPs among all hospitalized COVID-19 patients identified on the state level was slightly lower than in our earlier report involving a large single center cohort from the University Hospital in Krakow (26,3%) (26). Still, multiple reasons may contribute to the higher frequency of hospitalizations in patients with comorbidities, including diabetes. DPs were older, with a higher prevalence of comorbidities, including cardiovascular disorders, thus subjecting them to COVID-19 complications. Infections are generally more common in DPs and often occur with increased severity (27). Some aspects of immune response to infections, including lymphocyte response, macrophage and granulocyte function, may be impaired in DPs group (28). Some other potential mechanisms responsible for a higher susceptibility to severe COVID-19 in DPs may include predisposition to hyperinflammatory reaction, higher affinity of SARS-Cov-2 virus to cell membranes and decreased viral clearance (29).

Diabetes was a risk factor for in-hospital death in hospitalized COVID-19 patients in the large cohort examined in our study. This is in line with previous studies, showing that diabetes is an independent risk factor for disease severity and in-hospital death (10, 15, 16, 30–32). It should be noted that the prevalence of numerous comorbidities, including cardiovascular diseases, was higher in DP than in non-diabetic patients. To date, research efforts focused primarily on single-center cohorts of patients, with less common analyses of datasets from large healthcare initiatives, or on nationwide level, thus, making our report noteworthy (15, 16).

Older age has been reported as an important risk factor for COVID-19-related mortality (33), which was also, as expected, observed in our study. Notably, the biggest difference in in-hospital mortality between DPs and non-diabetic patients was observed in 40-64 age groups, which is a result similar to some other studies (10, 34). Our results specifically point to patients aged 60-69, as in this group the impact of diabetes on COVD-19 in-hospital death differed the most between the two examined groups. It is likely that diabetes may not further increase the risk of mortality in elderly patients as the advanced age itself is one of the strongest risk factors (34). Conversely, in the younger population groups, the role played by diabetes is more apparent. Similar observations were reported for male patients in a recent systematic review, with nearly 50% higher risk of death than in males than in females (33). These distinctions were previously attributed to differences in the function of the immune system, including counts of selected lymphocytes, lack of some regulatory genes that are located on the X chromosome (33), and age-related mosaic loss of chromosome Y in elderly males (35). Moreover, a recent study explained these disparities to some extent by social and contextual factors (36).

Uniquely, in our study we developed a model that enabled us to quantify the risk of mortality in the cohort of patients hospitalized due to COVID-19 in Poland. The main strength of this model is the fact that it was supplied with data from a large population.

Finally, limitations of this study should be discussed. First, this is a retrospective, observational study and, thus, prone to many biases related to this study design. For this reason, causative relationships cannot be claimed based on this research. Secondly, we aimed to investigate solely patients admitted to the hospital, thus, our results cannot be automatically extended on the entire population affected by COVID-19. As previous studies showed, around half of the deaths due to COVID-19 occurred in non-hospitalized persons, with the majority of these patients residing in long-term care facilities (16). In addition, our models could have been supplied with deficient data as the data investigated included only that reported by various health providers. We are not able to assure the data’s full credibility. As we used billing data, some of comorbidities may have not been reported as individual diagnoses, such as obesity, with surprisingly low frequency reported in our dataset. We also cannot verify the criteria that were used to diagnose reported disorders.

Another limitation is that National Health Fund data includes only insured people. i.e. 90% of citizens of Poland. While our data on COVID-19 considered both insured and uninsured people, this can cause a potential bias on the information concerning other comorbidities for uninsured people, as only health services related to COVID-19 were state-financed for them.

Moreover, we were not able to differentiate between type 1 and type 2 diabetes, as there some inconsistency regarding reporting these with an appropriate ICD-10 code (in Polish translation of ICD-10 classification code E11 refers to non-insulin dependent diabetes and in English version it refers to type 2 diabetes). Nevertheless, the same data gathering methodology is routinely utilized by the Polish National Health Fund to inform the decision making. Thus, we believe that its quality was adequate for this study.

To summarize, in this nationwide retrospective study, diabetes was associated with higher frequency of hospitalization and a higher risk of in-hospital death in the course of COVID-19, regardless of sex, age and some of selected comorbidities, including chronic kidney disease, heart failure, chronic ischemic heart disease, cardiac arrythmias and neoplasms. However, the relative risk attributed to diabetes differed significantly across the age groups and genders. This relative risk was particularly high in males and patients in their sixties. This was one of the largest datasets of hospitalized diabetic patients analyzed since the outbreak of the COVID-19 pandemic in 2019 in Poland. Our findings can inform individual clinicians’ decisions and public healthcare providers on the risk associated with COVID-19 for individual populations.

## Data availability statement

The data analyzed in this study is subject to the following licenses/restrictions: Raw data analyzed in the article is proprietary to National Health Fund, Warsaw, Poland. Requests to access these datasets should be directed to maciej.malecki@uj.edu.pl.

## Ethics statement

The studies involving human participants were reviewed and approved by Jagiellonian University Ethics Committee. Written informed consent for participation was not required for this study in accordance with the national legislation and the institutional requirements.

## Author contributions

MK, JH, PW, TK and MTM contributed to conception and design of the study. BK and KK organized the database. BK and KK performed the statistical analysis. MK wrote the first draft of the manuscript. JH, PW, TK, MTM wrote sections of the manuscript. All authors contributed to the article and approved the submitted version.
