# Corrigendum: Diabetes as a risk factor of death in hospitalized COVID-19 patients – an analysis of a National Hospitalization Database from Poland, 2020

**DOI:** 10.3389/fendo.2023.1228521

**Published:** 2023-07-03

**Authors:** Michal Kania, Beata Koń, Konrad Kamiński, Jerzy Hohendorff, Przemysław Witek, Tomasz Klupa, Maciej T. Malecki

**Affiliations:** ^1^ Department of Metabolic Diseases and Diabetology, Jagiellonian University Medical College, Krakow, Poland; ^2^ Department of Metabolic Diseases and Diabetology, University Hospital, Krakow, Poland; ^3^ Department of Analysis and Innovation, National Health Fund, Warsaw, Poland

**Keywords:** COVID-19, diabetes, mortality, epidemiology, modelling, propensity-score matching

In the published article, there was an error. The ‘Acknowledgement’ section was omitted when preparing the published version of the article. In this section we acknowledge the input made by a linguist when revising the manuscript during the review process. The new Acknowledgments section is written below.

The authors acknowledge the editorial assistance of Ms. Joana Mielko, School of Medicine in English, Jagiellonian University Medical College.

The authors apologize for this error and state that this does not change the scientific conclusions of the article in any way. The original article has been updated.

