# Supplementary material for: Diabetes as a risk factor of death in hospitalized COVID-19 patients – an analysis of a National Hospitalization Database from Poland, 2020
Source: Front Endocrinol (Lausanne). 2023 May 4;14:1161637. doi: 10.3389/fendo.2023.1161637 (PMC10194032; doi:10.3389/fendo.2023.1161637)
Supplement: Supplementary file 1 [file Table_1.docx]

**Supplementary material**

Table S1. List of ICD codes for diagnosis of comorbidities.

| Comorbidity | ICD-10 code |
| --- | --- |
| Alcohol abuse | F10, E24.4, E52, G31.2, G62.1, I42.6, K29.2, K70.0, K70.3, K70.9, T51, X45, X65, Y15, Z50.2, Z71.4, Z72.1 |
| Cardiac arrhythmias | I44.1, I44.2, I44.3, I45.6, I45.9, I47, I48, I49, R00.0, R00.1, R00.8, T82.1, Z45.0, Z95.0 |
| Chronic pulmonary disorders | I27.8, I27.9, J40-J47, J60-J67, J68.4, J70.1, J70.3 |
| Coagulopathies | D65, D66, D67, D68, D69.1, D69.3, D69.4, D69.5, D69.6 |
| Heart failure | I09.9, I11.0, I13.0, I13.2, I25.5, I42.0, I42.5, I42.6, I42.7,I42.8, I42.9, I43, I50 |
| Anaemia | D50, D51, D52, D53, D54, D55, D56, D57, D58, D59, D60, D61, D62, D63, D64 |
| Depression | F20.4, F31.3, F31.4, F31.5, F32, F33, F34.1, F41.2, F43.2 |
| Diabetes | E10, E11, E12, E13, E14 |
| Drug abuse | F11, F12, F13, F14, F15, F16, F18, F19, Z71.5, Z72.2 |
| Electrolyte imbalances | E22.2, E86, E87 |
| Arterial hypertension | I10, I11, I12, I13, I15 |
| Hypothyroidism | E00, E01, E02, E03, E89.0 |
| Hyperthyroidism | E05 |
| Other thyroid disorders | E06, E07 |
| Liver disorders | B18, I85, I86.4, I98.2, K70, K71.1, K71.3, K71.4, K71.5,K71.7, K72, K73, K74, K76.0, K76.2-K76.9, Z94.4 |
| Neoplasm, malignant | C00-C97, D00-D09 |
| Neoplasm, other | D10-D48 |
| Obesity | E66 |
| Other neurological disorders | G10-G13, G20-G22, G25.4, G25.5, G31.2, G31.8, G31.9, G32, G35, G36, G37, G40, G41, G93.1, G93.4, R47.0, R56 |
| Paralysis | G04.1, G11.4, G80.1, G80.2, G81, G82, G83.0, G83.1,G83.2, G83.3, G83.4, G83.9 |
| Peripheral artery disease | I70, I71, I73.1, I73.8, I73.9, I77.1, I79.0, I79.2, K55.1,K55.8, K55.9, K95.8, Z95.9 |
| Psychosis | F20, F22, F23, F24, F25, F28, F29, F30.2, F31.2, F31.5 |
| Pulmonary circulation disorders | I26, I27, I28.0, I28.8, I28.9 |
| Chronic kidney disease | I12.0, I13.1, N18, N19, N25.0, Z49.0, Z49.1, Z49.2, Z94.0,Z99.2 |
| Rheumatoid diseases | L94.0, L94.1, L94.3, M05, M06, M08, M12.0, M12.3, M30,M31.0, M31.1, M31.2, M31.3, M32-M35, M45, M46.1,M46.8, M46.9 |
| Valvular disorders | A52.0, I05-I08, I09.1, I09.8, I34-I39, Q23.0, Q23.1, Q23.2,Q23.3, Z95.2, Z95.3, Z95.4 |
| Weight loss | E40, E41, E42, E43, E44, E45, E46, R63.4, R64 |
| Dyslipidaemia | E78 |
| Angina | I20 |
| History of myocardial infarction | I21, I22 |
| Chronic ischemic heart disease | I25 |
| Cardiomyopathy | I42, I43 |
| History of stroke | I63, I64 |
| Atrial fibrillation | I48 |
| Haemodialysis | 39.95 |

Table S2. Mortality of hospitalized patients across all age groups and genders.

| Age group (years) | Gender | Number of patients | In-hospital deaths | Mortality |  | Age group (years) | Gender | Number of patients | In-hospital deaths | Mortality |
| --- | --- | --- | --- | --- | --- | --- | --- | --- | --- | --- |
| Non-diabetic patients | | | | |  | **Diabetic patients** | | | | |
| 18-24 | F | 2810 | 6 | 0.2% |  | 18-24 | F | 71 | 1 | 1.4% |
| 18-24 | M | 2444 | 5 | 0.2% |  | 18-24 | M | 40 | 0 | 0.0% |
| 25-34 | F | 8230 | 18 | 0.2% |  | 25-34 | F | 317 | 3 | 0.9% |
| 25-34 | M | 7500 | 45 | 0.6% |  | 25-34 | M | 156 | 6 | 3.9% |
| 35-44 | F | 8428 | 66 | 0.8% |  | 35-44 | F | 420 | 14 | 3.3% |
| 35-44 | M | 10117 | 151 | 1.5% |  | 35-44 | M | 581 | 37 | 6.4% |
| 45-54 | F | 8657 | 138 | 1.6% |  | 45-54 | F | 809 | 47 | 5.8% |
| 45-54 | M | 9738 | 337 | 3.5% |  | 45-54 | M | 1434 | 100 | 6.9% |
| 55-64 | F | 9846 | 405 | 4.1% |  | 55-64 | F | 2334 | 195 | 8.4% |
| 55-64 | M | 12894 | 984 | 7.6% |  | 55-64 | M | 4079 | 474 | 11.6% |
| 65-74 | F | 10907 | 930 | 8.5% |  | 65-74 | F | 5805 | 770 | 13.3% |
| 65-74 | M | 13994 | 1797 | 12.8% |  | 65-74 | M | 7998 | 1363 | 17.0% |
| 75-84 | F | 8912 | 1337 | 15.0% |  | 75-84 | F | 5974 | 1026 | 17.2% |
| 75-84 | M | 8485 | 1827 | 21.5% |  | 75-84 | M | 5056 | 1160 | 22.9% |
| 85+ | F | 7148 | 1592 | 22.3% |  | 85+ | F | 3325 | 808 | 24.3% |
| 85+ | M | 4343 | 1259 | 29.0% |  | 85+ | M | 1769 | 537 | 30.4% |
| F – female, M – male | | | | | | | | | | |

Table S3. Median estimates of the probability of COVID-19 in-hospital death - estimates from logistic regression with interactions.

| Age group | Non-diabetic patients | Diabetic patients |
| --- | --- | --- |
| 18-24 | 0.09% | 0.53% |
| 25-29 | 0.21% | 1.00% |
| 30-34 | 0.44% | 1.46% |
| 35-39 | 0.81% | 2.77% |
| 40-44 | 1.35% | 4.02% |
| 45-49 | 2.16% | 5.40% |
| 50-54 | 3.30% | 7.10% |
| 55-59 | 4.80% | 9.04% |
| 60-64 | 6.98% | 11.23% |
| 65-69 | 9.32% | 13.64% |
| 70-74 | 12.08% | 16.03% |
| 75-79 | 15.25% | 18.46% |
| 80-84 | 19.00% | 21.05% |
| 85-89 | 22.56% | 23.53% |
| 90-94 | 26.07% | 25.43% |
| 95+ | 29.58% | 27.18% |

Table S4. The characteristics of the hospitalized population after PSM analysis.

| **Characteristics** | **All** |  | **Diabetes** | **Non-diabetes** | **P value** |
| --- | --- | --- | --- | --- | --- |
| Number | 101 578 |  | 19 050 (18.8%) | 82 528 (81.2%) | - |
| Age >65 years [n (%)] | 38 592 (38%) |  | 13 117 (68.9%) | 25 475 (30.9%) | <0.001 |
| Age > 85 years [n (%)] | 6 637 (6.5%) |  | 2 121 (11.1%) | 4 516 (5.5%) | <0.001 |
| Male [n (%)] | 54 628 (53.8%) |  | 10 552 (55.2%) | 44 076 (53.4%) | <0.001 |
| **Endpoints** |  |  |  |  |  |
| In-hospital death [Yes (%)] | 7568 (7.5%) |  | 2756 (14.4%) | 4812 (5.8%) | <0.001 |

Table S5. The prevalence of comorbidities in the hospitalized population after PSM analysis.

| Comorbidities | Non-diabetic | Diabetic | P |
| --- | --- | --- | --- |
| Number | 82 858 (81.2%) | 19 050 (18.8%) |  |
| Arterial hypertension | 29 875 (36.2%) | 15 330 (80.5%) | <0.001 |
| Neoplasm, other | 10 440 (12.7%) | 2 806 (14.7%) | <0.001 |
| Heart failure | 5 265 (6.4%) | 3 974 (20.9%) | <0.001 |
| Cardiac arrhythmias | 5 645 (6.8%) | 3 198 (16.8%) | <0.001 |
| Dyslipidaemia | 8 773 (10.6%) | 4 993 (26.2%) | <0.001 |
| Chronic ischemic heart disease | 5 348 (6.5%) | 4 084 (21.4%) | <0.001 |
| Chronic pulmonary disorders | 6 652 (8.1%) | 2 423 (12.7%) | <0.001 |
| Peripheral artery disease | 4 209 (5.1%) | 2 768 (14.5%) | <0.001 |
| Atrial fibrillation | 3 433 (4.2%) | 2 351 (12.3%) | <0.001 |
| Neoplasm, malignant | 4 011 (4.9%) | 1 753 (9.2%) | <0.001 |
| Anaemia | 2 170 (2.6%) | 972 (5.1%) | <0.001 |
| Hypothyroidism | 3 375 (4.1%) | 1 141 (6%) | <0.001 |
| Chronic kidney disease | 1 155 (1.4%) | 1 044 (5.5%) | <0.001 |
| Depression | 2 451 (3%) | 646 (3.4%) | 0.002 |
| Other neurological disorders | 1 725 (2.1%) | (539 (2.8%) | <0.001 |
| History of stroke | 1 519 (1.8%) | 888 (4.7%) | <0.001 |
| Angina | 842 (1%) | 657 (3.5%) | <0.001 |
| Electrolyte imbalances | 768 (0.9%) | 459 (2.4%) | <0.001 |
| Alcohol abuse | 1 873 (2.3%) | 349 (1.8%) | <0.001 |
| Obesity | 1 073 (1.3%) | 813 (4.3%) | <0.001 |
| Liver disorders | 825 (1%) | 351 (1.8%) | <0.001 |
| Valvular disorders | 305 (0.4%) | 255 (1.3%) | <0.001 |
| Rheumatoid diseases | 693 (0.8%) | 272 (1.4%) | <0.001 |
| History of myocardial infarction | 437 (0.5%) | 343 (1.8%) | <0.001 |
| Other thyroid disorders | 635 (0.8%) | 179 (0.9%) | 0.016 |
| Paralysis | 380 (0.5%) | 246 (1.3%) | <0.001 |
| Psychosis | 734 (0.9%) | 183 (1%) | 0.322 |
| Hyperthyroidism | 124 (0.2%) | 86 (0.5%) | <0.001 |
| Dialysis | 190 (0.2%) | 137 (0.7%) | <0.001 |
| Pulmonary circulation disorders | 132 (0.2%) | 69 (0.4%) | <0.001 |
| Coagulopathies | 83 (0.1%) | 48 (0.3%) | <0.001 |
| Cardiomyopathy | 58 (0.1%) | 50 (0.3%) | <0.001 |
| Drug abuse | 157 (0.2%) | 36 (0.2%) | 0.992 |
| Weight loss | 50 (0.1%) | 29 (0.2%) | <0.001 |

Table S6. Mortality of hospitalized patients across all groups and genders after PSM analysis.

| Age group (years) | Gender | Number of patients | Mortality |  | Age group (years) | Gender | Number of patients | Mortality |
| --- | --- | --- | --- | --- | --- | --- | --- | --- |
| Non-diabetics | | | |  | Diabetic patients | | | |
| 18-24 | F | 2 073 | 0.10% |  | 18-24 | F | 49 | 0.00% |
| 18-24 | M | 1 745 | 0.00% |  | 18-24 | M | 31 | 0.00% |
| 25-34 | F | 6 713 | 0.07% |  | 25-34 | F | 254 | 0.00% |
| 25-34 | M | 5 681 | 0.46% |  | 25-34 | M | 108 | 1.85% |
| 35-44 | F | 5 968 | 0.34% |  | 35-44 | F | 267 | 2.25% |
| 35-44 | M | 7 749 | 0.95% |  | 35-44 | M | 382 | 4.71% |
| 45-54 | F | 5 431 | 0.90% |  | 45-54 | F | 428 | 3.97% |
| 45-54 | M | 7 212 | 2.68% |  | 45-54 | M | 918 | 5.77% |
| 55-64 | F | 5 922 | 2.63% |  | 55-64 | F | 1 179 | 6.11% |
| 55-64 | M | 8 559 | 6.64% |  | 55-64 | M | 2 317 | 10.88% |
| 65-74 | F | 5 416 | 7.13% |  | 65-74 | F | 2 497 | 11.05% |
| 65-74 | M | 8 060 | 11.34% |  | 65-74 | M | 4 050 | 15.63% |
| 75-84 | F | 3 784 | 13.72% |  | 75-84 | F | 2 333 | 16.59% |
| 75-84 | M | 3 699 | 21.36% |  | 75-84 | M | 2 116 | 22.54% |
| 85+ | F | 3 145 | 22.07% |  | 85+ | F | 1 491 | 24.88% |
| 85+ | M | 1 371 | 30.27% |  | 85+ | M | 630 | 30.48% |
| F – female, M – male | | | | | | | | |

Table S7. Median estimates of the probability of COVID-19 in-hospital death - estimates from logistic regression with interactions after PSM.

| Age group | Non-diabetic patients | Diabetic patients |
| --- | --- | --- |
| 18-24 | 0.04% | 0.25% |
| 25-29 | 0.09% | 0.56% |
| 30-34 | 0.22% | 0.78% |
| 35-39 | 0.46% | 1.78% |
| 40-44 | 0.87% | 2.84% |
| 45-49 | 1.54% | 4.36% |
| 50-54 | 2.54% | 6.13% |
| 55-59 | 3.97% | 7.90% |
| 60-64 | 5.93% | 10.72% |
| 65-69 | 8.51% | 12.97% |
| 70-74 | 11.76% | 16.10% |
| 75-79 | 15.28% | 18.15% |
| 80-84 | 18.73% | 20.52% |
| 85-89 | 21.44% | 23.25% |
| 90-94 | 24.71% | 26.09% |
| 95+ | 28.67% | 29.02% |
